# Association of Autism Spectrum Disorder, Neuroticism, and Subjective Well-Being With Cardiovascular Diseases: A Two-Sample Mendelian Randomization Study

**DOI:** 10.3389/fcvm.2021.676030

**Published:** 2021-06-11

**Authors:** Xingang Sun, Lu Chen, Zhen Wang, Yunlong Lu, Miao Chen, Yuxian He, Hongfei Xu, Liangrong Zheng

**Affiliations:** ^1^Department of Cardiology, The First Affiliated Hospital, School of Medicine, Zhejiang University, Hangzhou, China; ^2^Department of Cardiothoracic Surgery, The First Affiliated Hospital, School of Medicine, Zhejiang University, Hangzhou, China

**Keywords:** autism spectrum disorder, neuroticism, subjective well-being, mendelian randomization, causality

## Abstract

**Background:** Previous observational studies have reported an association between psychiatric traits and cardiovascular diseases (CVDs). In this two-sample Mendelian randomization (MR) study, we aimed to investigate the causality between psychiatric traits and CVDs.

**Methods:** Single-nucleotide polymorphisms (SNPs) associated with autism spectrum disorder (ASD), neuroticism, and subjective well-being at genome-wide significance (*P* < 1 × 10^−8^) were identified from genome-wide association studies. Summary-level data of the outcomes, including coronary artery disease (CAD), myocardial infarction (MI), atrial fibrillation (AF), and heart failure (HF), were obtained from several largest datasets. The inverse-variance weighted (IVW) method was used as our main analyses to conduct this MR study. Sensitivity analyses included the weighted median, the MR-robust adjusted profile score (MR-RAPS), and the MR pleiotropy residual sum and outlier (MR-PRESSO) method. Repeated MR analyses using a more relaxed threshold (*P* < 1 × 10^−6^) for instruments selection and multivariable MR analyses were also applied to evaluate the robustness of results.

**Results:** The MR analyses showed that genetic predisposition to ASD was associated with a higher risk of AF [odds ratio (OR), 1.109; 95% confidence interval (CI), 1.023–1.201; *P* = 0.011] and HF (OR, 1.138; 95% CI, 1.036–1.251; *P* = 0.007). Neuroticism was casually associated with an increased risk of AF (OR, 1.201; 95% CI, 1.037–1.392; *P* = 0.015), whereas subjective well-being had a protective effect on HF (OR, 0.732; 95% CI, 0.574–0.933; *P* = 0.012). No other causal association between psychiatric traits and CVDs was observed. Consistent results were obtained in sensitivity analyses.

**Conclusion:** This study provided evidence of causal associations of ASD with a higher risk of AF and HF. Besides, neuroticism was casually associated with an increased risk of AF, and subjective well-being was associated with a decreased risk of HF.

## Introduction

Common types of cardiovascular diseases (CVDs) include coronary artery disease (CAD), myocardial infarction (MI), atrial fibrillation (AF), and heart failure (HF). In America, ~840,000 people died due to CVDs in 2016 alone, and 120 million people suffer from more than one kind of CVDs currently (1, 2). More than that, CVDs remain the leading cause of morbidity and mortality and produce growing health and economic burdens worldwide for the aging of the population (3). Apart from traditionally recognized risk factors, such as smoking, obesity, lack of physical activity, hypertension, hyperlipidemia, hyperglycemia, etc. (4), observational studies suggested that several psychiatric traits are potential risk factors for CVDs.

A meta-analysis of 30 prospective studies suggested that depression was an independent risk factor for coronary heart disease (CHD) [relative risks (RR), 1.30; 95% confidence interval (CI), 1.22–1.40] and MI (RR, 1.30; 95% CI, 1.18–1.44) (5). The Guilford–Zimmerman Temperament Survey assessing the association between personality traits and longevity showed that emotional stability was significantly associated with the risk of death due to CVDs [hazard ratio (HR), 0.979; 95% CI, 0.960–0.999] (6). A case–control study also revealed that CVDs were more prevalent in adults with autism spectrum disorder (ASD) (7). Besides, neuroticism was indicated to be related to an increased risk of CVDs mortality (8). Taken together, these observations lead to a conclusion that psychiatric traits are closely related to CVDs; however, the causality remains unknown.

Mendelian randomization (MR) is an approach using genetic variants as instrumental variables (IVs) to explore the causal relationship between risk factors (exposures) and outcomes (9). Given the genetic variants randomly distributed at conception, MR studies are less susceptible to the confounding and reverse causation of conventional observational studies (10). Recently, this method has been used to estimate the causal relationship between depression and the risk of CVDs. Li et al. demonstrated that depression was causally associated with a higher risk of CAD [odds ratio (OR), 1.099; 95% CI, 1.031–1.170; *P* = 0.004] and MI (OR, 1.146; 95% CI, 1.070–1.228; *P* = 1.05 × 10^−4^) (11), which was consistent with other two MR studies (12, 13). However, there is limited or no MR study when it comes to other psychiatric traits. Only one MR study evaluating the causal relationship between subjective well-being and cardiometabolic health showed that a higher body mass index was associated with lower subjective well-being (14), without causal effect observed between subjective well-being and other cardiometabolic health measures (including CAD and MI).

In this study, we applied a two-sample MR approach to detect the causality of genetically determined psychiatric traits on several types of CVDs (CAD, MI, AF, and HF). As the causal association between depression and CVDs has been fully investigated, psychiatric traits including ASD, neuroticism, and subjective well-being were chosen as exposures in our study.

## Materials and Methods

### Data Sources and SNP Selection

We selected three psychiatric traits that have been shown to correlate with CVDs in observational studies, including ASD, neuroticism, and subjective well-being as exposures. Single-nucleotide polymorphisms (SNPs) for psychiatric traits were restricted at the level of genome-wide significance (*P* < 5 × 10^−8^) (15, 16). To choose valid instrumental SNPs, several steps were taken. First, we pruned these SNPs not to be in linkage disequilibrium (LD) (*r*^2^ < 0.001, window size = 10,000 kb) or absent from LD reference panel (EUR population) by the clump function in package TwosampleMR (17), with two SNPs (rs201910565, rs71190156) for ASD, six SNPs (rs34761973, rs4257287, rs148466862, rs6929812, rs72696282, rs28732100) for neuroticism, and nine SNPs (rs7218235, rs149866169, rs114658852, rs11665070, rs815753, rs4442212, rs677325, rs28732100, rs28687557) for subjective well-being removed from the study. Second, we excluded pleiotropic SNPs associated with potential confounders (*P* < 5 × 10^−8^) by searching the PhenoScanner database (Supplementary Table 1) (18). Finally, 7 SNPs for ASD, 27 SNPs for neuroticism, and 35 SNPs for subjective well-being remained as IVs for exposures. Information on these SNPs is detailed in Supplementary Table 2. We also calculated the F statistic to assess the strength of each SNP. An *F* statistic > 10 indicated that it was strong enough to avoid weak instrument bias according to the formula of $F=R^{2}\frac{n-2}{1-R^{2}}$, where *R*^2^ refers to the proportion of variance explained by IVs and n stands for the sample size (19).

For disease outcomes, the summary statistics were derived from the Coronary Artery Disease Genome-Wide Replication and Meta-analysis plus the Coronary Artery Disease Genetics (CardiogramplusC4D) consortium for CAD and MI (20). As for AF, we obtained the summary statistics data from the genome-wide association studies (GWAS) performed by Nielsen et al. which involved 60,620 atrial fibrillation cases and 970,216 controls (21). For HF, the summary-level data were extracted from the largest GWAS meta-analysis among European individuals performed by the Heart Failure Molecular Epidemiology for Therapeutic Targets (HERMES) Consortium (22). If SNPs were not available in the outcome datasets, proxy SNPs (*r*^2^ > 0.8) were found to replace them by searching an online website (http://snipa.helmholtz-muenchen.de/snipa3/) based on the European population reference data from the 1,000 Genomes Project (23). Detailed information about data sources of psychiatric traits and CVDs are presented in Table 1. All data included in our study were available in public GWAS datasets; as a result, no specific ethical review approval or informed consent was required.

**Table 1:** Description of data sources included in the MR analyses.

**Trait**	**Data source**	**Sample size (cases/controls)**	**Population**
ASD (15)	PGC	18,381/27,969	European
Neuroticism (16)	UKB, GPC	168,105	European
Subjective well-being (16)	UKB, 23andMe, SSGAC	388,538	European
CAD/MI (20)	CARDIoGRAMplusC4D	60,801/123,504	Mixed (77% European)
AF (21)	Nielsen et al.	60,620/970,216	Mixed (98.6% European)
HF (22)	HERMES	47,309/930,014	European
Body mass index (30)	Hoffmann et al.	334,487	Mixed (94.3% European)
Hypertension, SBP, DBP	Neale laboratory	317,754	European

*ASD, autism spectrum disorder; CAD, coronary artery disease; MI, myocardial infarction; AF, atrial fibrillation; HF, heart failure; PGC, the Psychiatric Genomics Consortium; UKB, the UK biobank; GPC, the Genetics of Personality Consortium; 23andMe, 23andMe company; SSGAC, Social Science Genetics Association Consortium; CARDIoGRAMplusC4D, Coronary Artery Disease Genome-Wide Replication and Meta-analysis plus the Coronary Artery Disease Genetics Consortium; HERMES, Heart Failure Molecular Epidemiology for Therapeutic Targets Consortium; SBP, systolic blood pressure; DBP, diastolic blood pressure*.

### Statistical Analysis

A two-sample MR approach was used in the present study. After harmonizing the data to ensure the effect of IVs on the exposures and the outcomes corresponding to the same allele, we obtained effect estimates of genetically predicted exposures on outcomes using the fixed-effects inverse-variance weighted (IVW) method as our main analyses. Several sensitivity analyses, including the weighted median method (24), the MR-robust adjusted profile score (MR-RAPS) (25), and the MR pleiotropy residual sum and outlier (MR-PRESSO) (26) method were carried out. The weighted median method assumed that at least half of the weight were from valid variants, providing consistent estimates on causal effects (24). The MR-RAPS corrected for horizontal pleiotropy in the IVW analyses by using robust adjusted profile scores (25). The MR-PRESSO method was used to conduct a global test of heterogeneity to detect and correct for horizontal pleiotropic outliers in the IVW method (26). Apart from the above, several pleiotropy assessments were undertaken to evaluate the robustness of the results. First, heterogeneity statistics were calculated by Cochran's Q statistic. A Cochran's Q-derived *P* < 0.05 was considered as heterogeneity. If there was significant heterogeneity, a random-effects IVW model would be implemented, which was less prone to the bias of weaker SNP-exposure associations (27). Second, the intercept test from MR-Egger was also applied, and a zero intercept from MR-Egger indicated an absence of direct effects of SNPs on the outcome not via the exposure (horizontal pleiotropy) (28). Third, scatter plots depicting the relationship of the SNP effects on the exposure against the outcome were also provided. Lastly, leave-one-out analyses were applied to evaluate the stability of effect sizes and to identify whether the casual results were driven by any individual SNP.

Considering the low number of variants used in our MR study especially for ASD, genetic variants with higher *P* values for exposures (*P* < 1 × 10^−6^) were selected by the same steps stated above and then repeated the MR analyses to evaluate whether the significant results were robust. Details on the valid IVs (*P* < 1 × 10^−6^) can be found in Supplementary Table 3. In addition, noticing that the pleiotropic SNPs identified by searching the PhenoScanner database were mainly associated with body mass index (BMI) and blood pressure, we therefore performed regression-based multivariable MR to obtain estimates that were independent of effects of BMI and blood pressure (29). Summary-level data for genetic association of IVs with BMI were available from a large meta-analysis of GWAS conducted by Hoffmann et al. (30), and blood pressure measurements (hypertension, systolic, and diastolic blood pressure) were obtained from UK Biobank, which was published by Neale laboratory (http://www.nealelab.is/uk-biobank/). Detailed characteristics about these sources can be found in Table 1.

Power calculations were performed to evaluate the required effect of exposure on the outcome at 80% power according to the sample size of each outcome and variance of exposures explained by the genetic variants on a web-based application (http://cnsgenomics.com/shiny/mRnd/), and results are displayed in Supplementary Table 4. Odds ratios (ORs) were scaled per standard deviation increment in exposures. All statistical analyses were two-sided and considered statistically significant at *P* < 0.05. The package TwosampleMR (17), MendelianRandomization (31), and MR-PRESSO (26) in R (Version 4.0.2) were used to conduct the MR analyses.

## Results

### Autism Spectrum Disorder

**Figure 1:**
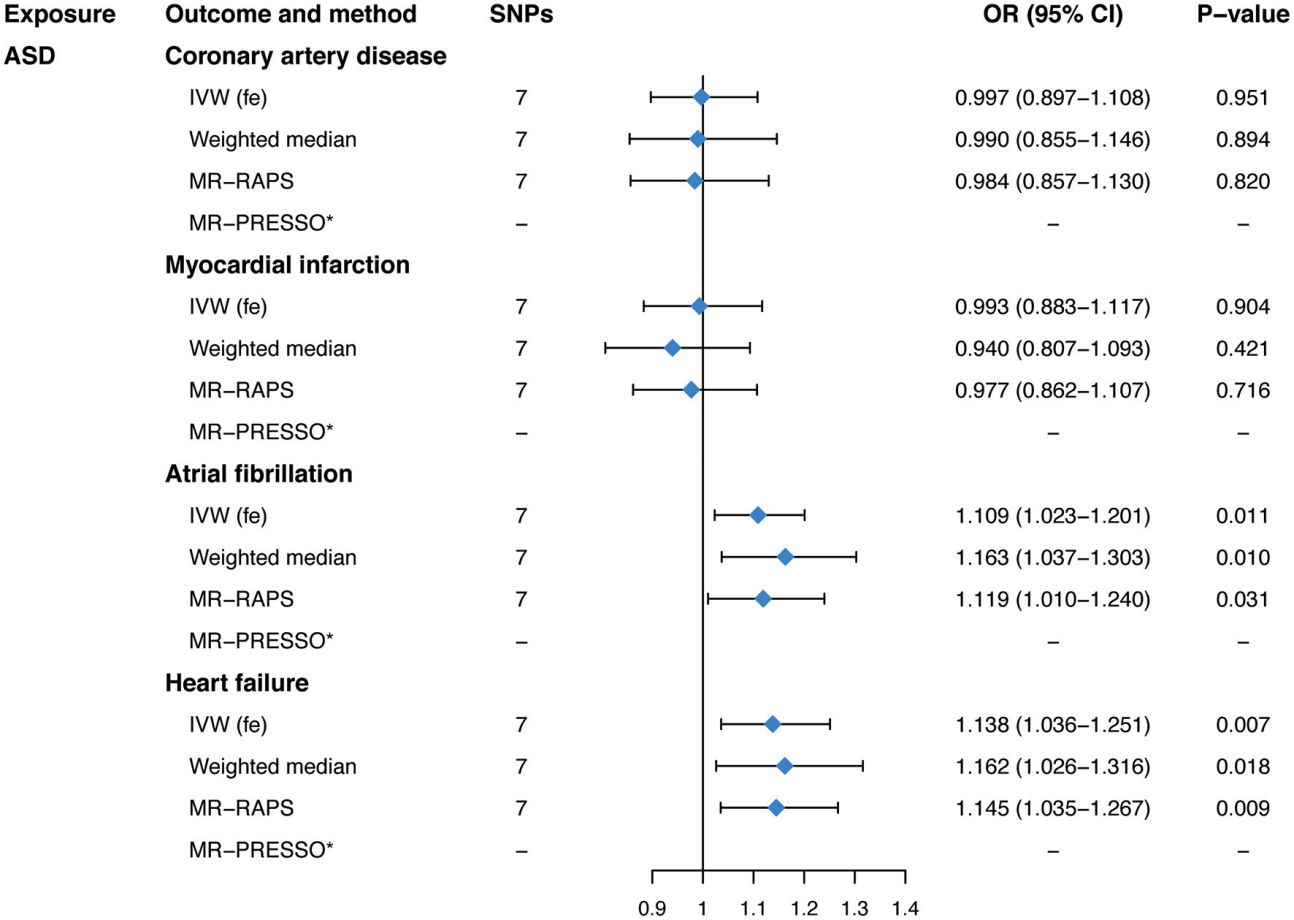
Mendelian randomization estimates of genetically predicted autism spectrum disorder on four cardiovascular diseases. SNPs, single-nucleotide polymorphisms; OR, odds ratio; CI, confidence interval; ASD, autism spectrum disorder; IVW (fe), fixed-effects inverse-variance weighted; MR-RAPS, Mendelian randomization-robust adjusted profile score; MR-PRESSO, Mendelian randomization pleiotropy residual sum and outlier.

In the standard IVW analyses, genetic predisposition to ASD was associated with a higher risk of AF (OR, 1.109; 95% CI, 1.023–1.201; *P* = 0.011; Figure 1) and HF (OR, 1.138; 95% CI, 1.036–1.251; *P* = 0.007; Figure 1). However, ASD was not casually associated with CAD (OR, 0.997; 95% CI, 0.897–1.108; *P* = 0.951; Figure 1) or MI (OR, 0.993; 95% CI, 0.883–1.117; *P* = 0.904; Figure 1). Similar findings were observed across sensitivity analyses (Figure 1). The Cochran's Q statistics showed no evidence of heterogeneity in the IVW analyses (*P* = 0.266 for CAD, *P* = 0.408 for MI, *P* = 0.059 for AF, *P* = 0.564 for HF, respectively; Table 2). Whereas, there was evidence of directional pleiotropy for CAD (*P* for intercept = 0.049; *P* for global test = 0.224; Table 2) but not for MI, AF, or HF (*P* for intercept >0.170; P for global test >0.06; Table 2). Deletion of IV rs910805 and rs10099100 had modest influences on the causal relationship between ASD and AF (Supplementary Figure 1). Furthermore, rs910805 slightly influenced the causal inference results of ASD and HF (Supplementary Figure 2). After repeating the MR analyses by adopting a more relaxed *P*-value threshold, ASD remained significantly associated with HF in the fixed-effects IVW method and sensitivity analyses without any evidence of pleiotropy (Supplementary Tables 5, 6). Although the relationship between ASD and AF was significant in the fixed-effects IVW method, it was not consistent across sensitivity analyses (Supplementary Tables 5, 6).

**Table 2:** Potential pleiotropy evaluation using different methods.

**Exposure**	**Outcome**	**SNPs**	**Cochran's Q statistic**	**Cochran's Q**	**MR-Egger intercept**	**MR-PRESSO global test**	**MR-PRESSO global test**
				***P***	***P***		***P***
ASD	CAD	7	7.639	0.266	0.049	11.399	0.224
	MI	7	6.135	0.408	0.176	7.966	0.476
	AF	7	12.115	0.059	0.170	16.588	0.060
	HF	7	4.842	0.564	0.687	7.027	0.562
Neuroticism	CAD	27	48.787	0.004	0.762	52.507	0.002
	CAD^*^	26	34.254	0.103	0.357	36.962	0.095
	MI	27	43.794	0.016	0.665	47.159	0.011
	MI^*^	26	36.102	0.070	0.795	39.079	0.080
	AF	27	24.496	0.548	0.954	26.396	0.575
	HF	27	34.009	0.135	0.176	36.621	0.144
Subjective well-being	CAD	35	39.487	0.238	0.572	41.944	0.256
	MI	35	34.201	0.458	0.459	36.325	0.429
	AF	35	35.257	0.409	0.264	37.560	0.402
	HF	35	47.334	0.064	0.835	49.931	0.075

*SNP, single-nucleotide polymorphism; MR-Egger, Mendelian randomization-Egger; MR-PRESSO, Mendelian randomization pleiotropy residual sum and outlier; ASD, autism spectrum disorder; CAD, coronary artery disease; MI, myocardial infarction; AF, atrial fibrillation; HF, heart failure*.

* *Excluding the outliers for CAD (rs1400867) and MI (rs9427672), respectively, and using remained instrumental SNPs to perform pleiotropy assessment*.

### Neuroticism

**Figure 2:**
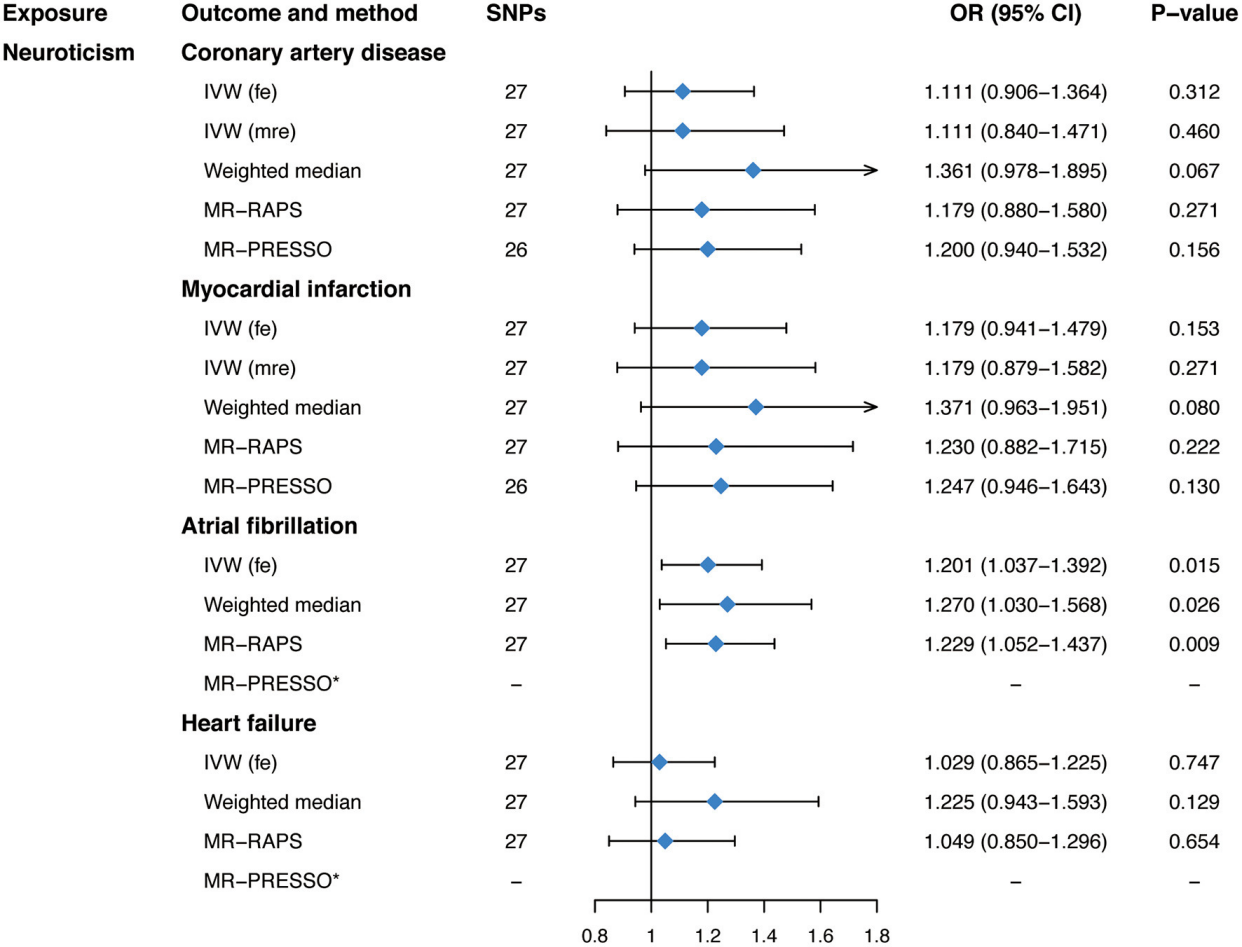
Mendelian randomization estimates of genetically predicted neuroticism on four cardiovascular diseases. SNPs, single-nucleotide polymorphisms; OR, odds ratio; CI, confidence interval; IVW (fe), fixed-effects inverse-variance weighted; IVW (mre), multiplicative random-effects inverse-variance weighted; MR-RAPS, Mendelian randomization-robust adjusted profile score; MR-PRESSO, Mendelian randomization pleiotropy residual sum and outlier.

Genetically predicted neuroticism was casually associated with an increased risk of AF (OR, 1.201; 95% CI, 1.037–1.392; *P* = 0.015; Figure 2) in the fixed-effects IVW method. Whereas, no causal association between neuroticism and CAD (OR, 1.111; 95% CI, 0.906–1.364; *P* = 0.312; Figure 2), MI (OR, 1.179; 95% CI, 0.941–1.479; *P* = 0.153; Figure 2), or HF (OR, 1.029; 95% CI, 0.865–1.225; *P* = 0.747; Figure 2) was observed in our main analyses. Sensitivity analyses obtained similar effects of neuroticism on CVDs (Figure 2), as well as causal estimates of neuroticism on AF in the leave-one-out analysis and scatter plots (Supplementary Figure 3). However, there was evidence of heterogeneous SNPs for CAD and MI (*P* < 0.016, Table 2) except for AF and HF (*P* > 0.135, Table 2), suggesting that the fixed-effects IVW estimates for CAD/MI might be invalid. Thus, we applied the random-effects IVW method, which still showed no significant causal association of neuroticism with CAD or MI (Figure 2). The MR-Egger regression for all outcomes was close to zero (*P* for intercept >0.176, Table 2), suggesting that there was no indication of directional pleiotropy. The MR-PRESSO method identified one outlier for CAD (rs1400867) and one outlier for MI (rs9427672). Exclusion of these outliers, respectively, did not essentially change the results for CAD or MI (Figure 2), with *P*-value for heterogeneity and MR-PRESSO global test becoming not significant anymore (*P* > 0.070, Table 2). Although the effect estimate was directionally consistent and no pleiotropy was indicated, the casual association between neuroticism and AF was not robust to the *P*-value threshold of 1 × 10^−6^ (Supplementary Tables 5, 6).

### Subjective Well-Being

**Figure 3:**
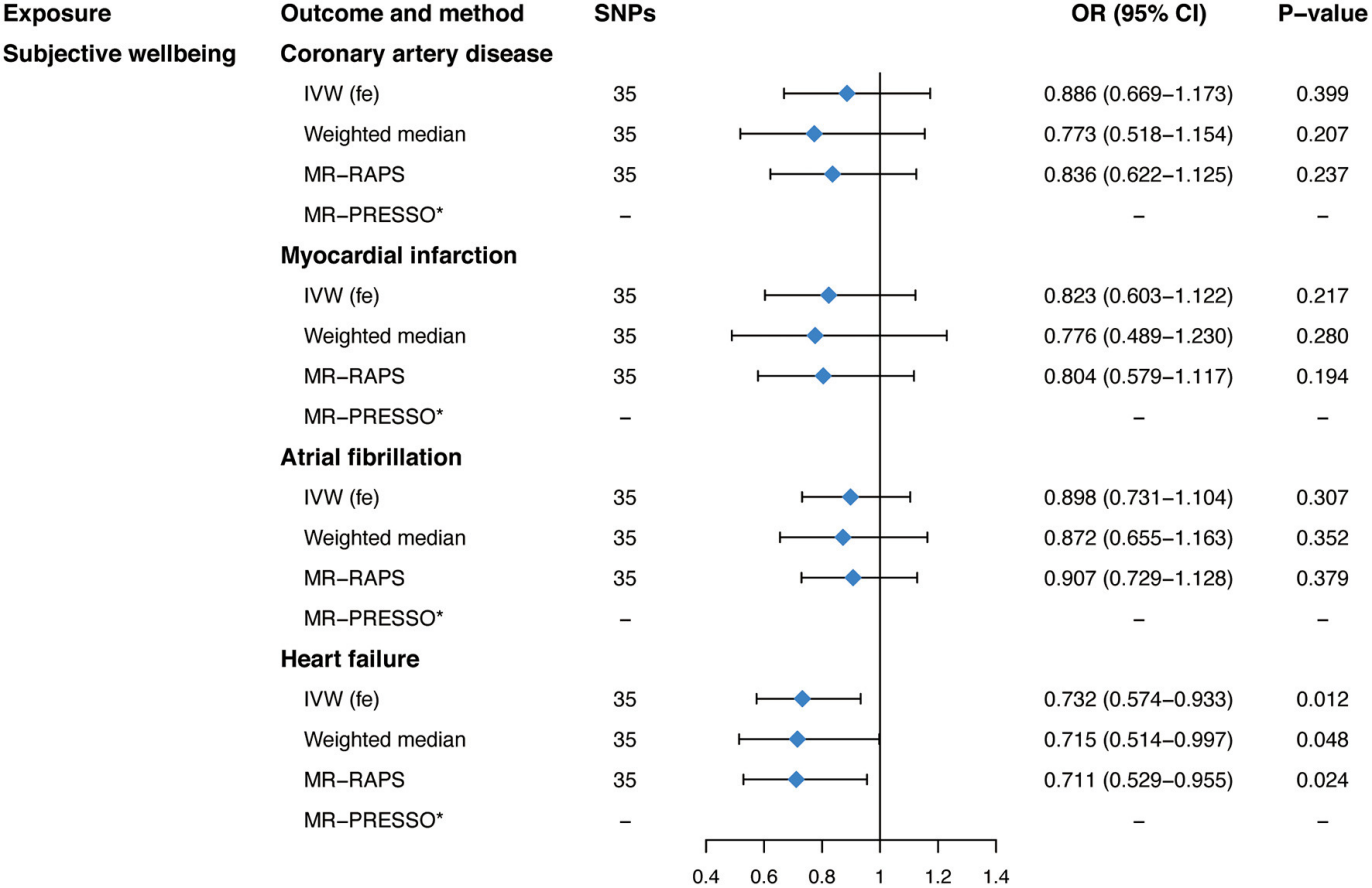
Mendelian randomization estimates of genetically predicted subjective well-being on four cardiovascular diseases. SNPs, single-nucleotide polymorphisms; OR, odds ratio; CI, confidence interval; IVW (fe), fixed-effects inverse-variance weighted; IVW (mre), multiplicative random-effects inverse-variance weighted; MR-RAPS, Mendelian randomization-robust adjusted profile score; MR-PRESSO, Mendelian randomization pleiotropy residual sum and outlie.

Our main analyses, as shown in Figure 3, suggested a protective effect of genetically predicted subjective well-being on HF (OR, 0.732; 95% CI, 0.574–0.933; *P* = 0.012) using the fixed-effects IVW analyses. However, there was no significant association between subjective well-being and CAD (OR, 0.886; 95% CI, 0.669–1.173; *P* = 0.399), MI (OR, 0.823; 95% CI, 0.603–1.122; *P* = 0.217), or AF (OR, 0.898; 95% CI, 0.731–1.104; *P* = 0.307). MR estimates were robust and consistent in sensitivity analyses, including the weighted median and the MR-RAPS method. No evidence of heterogeneity was observed as measured by Cochran's Q (*P* = 0.238 for CAD, *P* = 0.458 for MI, *P* = 0.409 for AF, *P* = 0.064 for HF, respectively; Table 2). Importantly, the intercept of the MR-Egger method and the MR-PRESSO global test also confirmed the absence of pleiotropy (*P* for intercept >0. 264; *P* for global test >0.075; Table 2). Furthermore, the leave-one-out analysis and scatter plots indicated that the causal effect of subjective well-being on HF was not driven by any individual SNP (Supplementary Figure 4). Moreover, when it comes to a more relaxed threshold (*P* < 1 × 10^−6^) for instruments selection, consistent protective effect of subjective well-being on HF was obtained in the fixed-effects IVW method as well as in sensitivity analyses, although there was some evidence of pleiotropy (Supplementary Tables 5, 6).

### Multivariable MR

Multivariable MR was performed to evaluate the robustness of significant results. Results indicated that the association of genetically predicted ASD with HF, genetically predicted neuroticism with AF, and genetically predicted subjective well-being with HF remained robust in the multivariable MR analyses adjusted for genetically determined body mass index or blood pressure separately (Table 3). However, the casual relationship between ASD and AF was attenuated due to wider CIs after adjusting for BMI or systolic or diastolic blood pressure (Table 3).

**Table 3:** Multivariable Mendelian randomization associations of autism spectrum disorder, neuroticism, and subjective well-being with atrial fibrillation and heart failure risk adjusting for body mass index and blood pressure measurements.

**Outcome/Model**	**OR (95% CI)**	***P***	**OR (95% CI)**	***P***
**AF**	**ASD**		**Neuroticism**	
Unadjusted model	1.109 (1.023–1.201)	0.011	1.201 (1.037–1.392)	0.015
Adjusted for BMI	1.126 (0.968–1.310)	0.124	1.209 (1.043–1.401)	0.012
Adjusted for hypertension	1.133 (1.011–1.270)	0.031	1.197 (1.034–1.387)	0.017
Adjusted for SBP	1.082 (0.944–1.241)	0.261	1.176 (1.058–1.309)	0.003
Adjusted for DBP	1.091 (0.961–1.239)	0.180	1.171 (1.058–1.298)	0.002
**HF**	**ASD**		**Subjective well-being**	
Unadjusted model	1.138 (1.036–1.251)	0.007	0.732 (0.574–0.933)	0.012
Adjusted for BMI	1.129 (1.005–1.267)	0.041	0.765 (0.586–0.997)	0.047
Adjusted for hypertension	1.156 (1.047–1.276)	0.004	0.737 (0.551–0.987)	0.041
Adjusted for SBP	1.176 (1.058–1.309)	0.003	0.742 (0.554–0.991)	0.044
Adjusted for DBP	1.171 (1.058–1.298)	0.002	0.729 (0.551–0.964)	0.027

*OR, odds ratio; CI, confidence interval; AF, atrial fibrillation; ASD, autism spectrum disorder; HF, heart failure; BMI, body mass index; SBP, systolic blood pressure; DBP, diastolic blood pressure*.

## Discussion

In this study, we employed a two-sample MR approach to explore the causality of ASD, neuroticism, and subjective well-being on CVDs (CAD, MI, AF, and HF). Our analyses suggested that genetically predicted ASD had risk effects on AF and HF, and neuroticism was related to a higher AF risk. Evidence also indicated a protective effect of subjective well-being on HF. No other causal association was observed.

The association between ASD and CVDs was controversial in previous observational studies. As shown in a Danish nationwide registry study, circulatory system diseases were less prevalent in the ASD group as compared with the control group (OR, 0.5; 95% CI, 0.3–1.1; *P* = 0.09) (32). Specifically, the occurrence of ischemic heart diseases was significantly lower in the ASD group (0 vs. 4.8%; *P* = 0.02) (32). However, a case–control study indicated that adults with ASD were more likely to be diagnosed with hyperlipidemia (OR, 2.0; 95% CI, 1.2–3.4, *P* = 0.012) (33), which is a major risk factor for CVDs. Another study, involving 1,507 adults with ASD and 15,070 controls, also suggested that CVDs were significantly more common in adults with ASD (OR, 2.54; 99% CI, 2.13–3.02; *P* < 0.001) (7). In our analyses, ASD was causally associated with a higher risk of AF (OR, 1.109; 95% CI, 1.023–1.201; *P* = 0.011) and HF (OR, 1.138; 95% CI, 1.036–1.251; *P* = 0.007). Although the precise mechanism linking ASD to CVDs was not well-elucidated, many lifestyle-related factors might be involved, such as atypical eating behavior, limited physical activity, and sedentary behavior (34, 35). As previous studies reported, people with ASD had higher rates of overweight and obesity than normative samples (36).

When it comes to neuroticism, the literature regarding the association between neuroticism and CVDs was limited. A 21-year prospective cohort study found that high neuroticism was related to an increased risk of CVDs mortality (HR, 1.12; 95% CI, 1.03–1.21) (8). This study further suggested that sociodemographic, health behavior, and physiological factors might contribute to this effect. However, another prospective study claimed that the association between neuroticism and cardiovascular mortality might differ as a function of socioeconomic status (37). It revealed that neuroticism was a risk factor for cardiovascular mortality in women with low socioeconomic status (HR, 2.02; 95% CI, 1.45–2.80); however, in higher socioeconomic status, it was protective (HR, 0.61; 95% CI, 0.38–0.97). Recently, Kranert et al. studied the association between AF-related symptom burden and personality traits and concluded that neuroticism was a strong independent predictor for symptomatic AF (38). This conclusion was similar to our findings that neuroticism was causally related to a higher risk of AF (OR, 1.201; 95% CI, 1.037–1.392; *P* = 0.015). As a known independent risk factor for cardiac mortality, decreased heart rate variability might explain such an effect to some extent. Riese et al. reported a negative correlation of neuroticism to heart rate variability (39). Furthermore, Cukić et al. also suggested that higher neuroticism was associated with reduced heart rate variability both under rest and stress (40).

Subjective well-being commonly refers to feelings of happiness or of life satisfaction (41, 42). Over the last decades, observational studies concluded that subjective well-being might be protective for CVDs. A Japanese large prospective study of middle-aged residents suggested that men with a low perceived level of life enjoyment showed an increased risk of total CVDs mortality (HR, 1.61; 95% CI, 1.32–1.96) (43). According to Boehm et al., satisfaction was significantly associated with a modestly reduced risk of total CHD (HR, 0.87; 95% CI, 0.78–0.98) (44). Another prospective study also concluded that positive psychological well-being (emotional vitality and optimism, with HR, 0.74; 95% CI, 0.55–0.98; and HR, 0.73; 95% CI, 0.54–0.99, respectively) was associated with reduced CHD risk (45). As for the possible mechanisms underlying the association between subjective well-being and CVDs, biological (autonomic, neuroendocrine, and inflammatory processes) and behavioral factors (physical activity, sleep quality and quantity, stress buffering, food consumption, smoking, and alcohol drinking) have been considered (43, 46). Recently, Wootton et al. conducted a bidirectional MR study to evaluate the causal association of subjective well-being on cardiometabolic health traits with 84 SNPs at *P* < 5 × 10^−5^ level and found no evidence of causality between subjective well-being and CAD or MI, in either direction (14). In line with this, our MR analyses did not support a causal relationship between subjective well-being and CAD or MI as well; however, a causal correlation was present between subjective well-being and HF (OR, 0.732; 95% CI, 0.574–0.933; *P* = 0.012). Our MR analyses extended the research of Wootton et al. by selecting SNPs at a more stringent threshold of *P* < 5 × 10^−8^ with 35 SNPs as IVs and including two other CVDs (AF and HF) as outcomes, indicating that our results were more credible. The inconsistency between observational studies and MR studies may be in part explained by the limitations of observational studies, such as possible confounding factors (including education, income, life circumstance, and nations) and reverse causality (47).

There are several implications for the clinical practice of our findings. Since psychiatric disorders, such as ASD, neuroticism, and depression, are potential risk factors for CVDs, high-quality clinical trials are warranted to estimate the ranking position of these psychiatric traits compared to classical risk factors for CVDs and the effectiveness of mental health treatments on reducing the risk of CVDs. Considering the large proportion of individuals with psychiatric disorders who also develop CVDs, early interventions within this group to improve physical health may represent an effective prevention strategy. Given that several lifestyle-related factors, such as physical activity, food consumption, smoking, and alcohol drinking, potentially mediate the pathway of psychiatric traits to CVDs, mental health management with a change in lifestyle-related factors may be needed to prevent CVDs in patients with psychiatric disorders. In addition, clinicians should detect psychiatric abnormality in patients with CVDs and choose effective therapies that address mental health but do not aggravate the underlying cardiovascular disorders. Furthermore, healthcare systems should be concerned to improve the well-being of the population for its protective effect on CVDs.

## Strengths and Limitations

Our study included several notable strengths. First, we used two-sample MR analyses to comprehensively assess the causal associations of three psychiatric traits with a broad range of CVDs. By using randomly allocated genetic variants as IVs, we minimized the bias of conventional confounders and reverse causality compared with observational studies. Moreover, summary statistics data derived from several largest GWAS datasets increased the precision of the SNPs selection and the statistical power of the analyses, as the casual effect sizes were confirmed close to or above the threshold of 80% statistical power. Finally, our conclusions were conducted based upon comprehensive analyses involving several reliable MR approaches and several pleiotropy assessments to avoid possible pleiotropic bias. Yet, several limitations deserved consideration. First, because the majority of the participants were of European ancestry, which reduced the bias from population stratification, our findings are less generalizable to other ancestries. Second, there was an indication of pleiotropy across SNPs in our analyses; nevertheless, best efforts were made to reduce the impact of pleiotropic bias. Third, we did not investigate sex-specific or socioeconomic-status-specific causal effects between three psychiatric traits and CVDs due to the lack of patient-level data. It was reported that perceived level of life enjoyment was associated with CVDs incidence and mortality in men, but not in women, suggesting that the causal relationship of psychiatric traits with CVDs might differ in different sexes and socioeconomic status, which was mentioned before (37, 43). Last, we did not apply the MR-Egger method for too broad CIs, consistent with other MR analyses involving psychiatric traits, which might lead to an inaccurate estimate (14, 48).

## Conclusion

In conclusion, our two-sample MR study suggested that genetic predisposition to ASD had risk effects on AF and HF, and neuroticism was correlated with an increased risk of AF. We also demonstrated that genetically predicted subjective well-being was a protective factor for HF. Further research focusing on the relationship of other psychiatric traits with CVDs are warranted, and the underlying mechanisms remain to be elucidated.

## Data Availability Statement

The raw data supporting the conclusions of this article will be made available by the authors, without undue reservation.

## Author Contributions

XS and LZ: conceptualization. XS and LC: methodology and writing—original draft preparation. LC, ZW, YL, MC, and YH: formal analysis. ZW, YL, MC, YH, LZ, and HX: writing—review and editing. LZ and HX: supervision and funding acquisition. All authors have read and agreed to the published version of the manuscript.

## Conflict of Interest

The authors declare that the research was conducted in the absence of any commercial or financial relationships that could be construed as a potential conflict of interest.

## References

[1] Roger VL Sidney S Fairchild AL Howard VJ Labarthe DR Shay CM. Recommendations for cardiovascular health and disease surveillance for 2030 and beyond: a policy statement from the American Heart Association. Circulation. (2020) 141:e104–19. 10.1161/CIR.0000000000000756 31992050

[2] Benjamin EJ Muntner P Alonso A Bittencourt MS Callaway CW Carson AP. Heart disease and stroke statistics-2019 update: a report from the American Heart Association. Circulation. (2019) 139: e56–528. 10.1161/CIR.0000000000000659 30700139

[3] Reddy KS Prabhakaran D. Reducing the risk of cardiovascular disease: brick by BRICS. Circulation. (2020) 141:800–2. 10.1161/CIRCULATIONAHA.119.044757 32150471

[4] Shen C Ge J. Epidemic of cardiovascular disease in China: current perspective and prospects for the future. Circulation. (2018) 138:342–4. 10.1161/CIRCULATIONAHA.118.033484 30571361

[5] Gan Y Gong Y Tong X Sun H Cong Y Dong X. Depression and the risk of coronary heart disease: a meta-analysis of prospective cohort studies. BMC Psychiatry. (2014) 14:371. 10.1186/s12888-014-0371-z 25540022 PMC4336481

[6] Terracciano A Löckenhoff CE Zonderman AB Ferrucci L Costa PT. Personality predictors of longevity: activity, emotional stability, and conscientiousness. Psychosom Med. (2008) 70:621–7. 10.1097/PSY.0b013e31817b9371 18596250 PMC2505356

[7] Croen LA Zerbo O Qian Y Massolo ML Rich S Sidney S. The health status of adults on the autism spectrum. Autism. (2015) 19:814–23. 10.1177/1362361315577517 25911091

[8] Shipley BA Weiss A Der G Taylor MD Deary IJ. Neuroticism, extraversion, and mortality in the UK Health and Lifestyle Survey: a 21-year prospective cohort study. Psychosom Med. (2007) 69:923–31. 10.1097/PSY.0b013e31815abf83 17991814

[9] Emdin CA Khera AV Kathiresan S. Mendelian randomization. JAMA. (2017) 318:1925–6. 10.1001/jama.2017.17219 29164242

[10] Georgiopoulos G Ntritsos G Stamatelopoulos K Tsioufis C Aimo A Masi S. The relationship between blood pressure and risk of atrial fibrillation: a Mendelian randomization study. Eur J Prev Cardiol. (2021). 10.1093/eurjpc/zwab005. [Epub ahead of print]. 33556963

[11] Li GH-Y Cheung C-L Chung AK-K Cheung BM-Y Wong IC-K Fok MLY. Evaluation of bi-directional causal association between depression and cardiovascular diseases: a Mendelian randomization study. Psychol Med. (2020). 10.1017/S0033291720003566. [Epub ahead of print]. 33032663

[12] Lu Y Wang Z Georgakis MK Lin H Zheng L. Genetic liability to depression and risk of coronary artery disease, myocardial infarction, and other cardiovascular outcomes. J Am Heart Assoc. (2021) 10:e017986. 10.1161/JAHA.120.017986 33372528 PMC7955472

[13] Tang B Yuan S Xiong Y He Q Larsson SC. Major depressive disorder and cardiometabolic diseases: a bidirectional Mendelian randomisation study. Diabetologia. (2020) 63:1305–11. 10.1007/s00125-020-05131-6 32270255 PMC7286869

[14] Wootton RE Lawn RB Millard LAC Davies NM Taylor AE Munafò MR. Evaluation of the causal effects between subjective wellbeing and cardiometabolic health: mendelian randomisation study. BMJ. (2018) 362:k3788. 10.1136/bmj.k3788 30254091 PMC6155050

[15] Grove J Ripke S Als TD Mattheisen M Walters RK Won H. Identification of common genetic risk variants for autism spectrum disorder. Nat Genet. (2019) 51:431–44. 10.1038/s41588-019-0344-8 30804558 PMC6454898

[16] Turley P Walters RK Maghzian O Okbay A Lee JJ Fontana MA. Multi-trait analysis of genome-wide association summary statistics using MTAG. Nat Genet. (2018) 50:229–37. 10.1038/s41588-017-0009-4 29292387 PMC5805593

[17] Hemani G Zheng J Elsworth B Wade KH Haberland V Baird D. The MR-Base platform supports systematic causal inference across the human phenome. Elife. (2018) 7:e34408. 10.7554/eLife.34408 29846171 PMC5976434

[18] Kamat MA Blackshaw JA Young R Surendran P Burgess S Danesh J. PhenoScanner V2: an expanded tool for searching human genotype-phenotype associations. Bioinformatics. (2019) 35:4851–3. 10.1093/bioinformatics/btz469 31233103 PMC6853652

[19] Pierce BL Ahsan H Vanderweele TJ. Power and instrument strength requirements for Mendelian randomization studies using multiple genetic variants. Int J Epidemiol. (2011) 40:740–52. 10.1093/ije/dyq151 20813862 PMC3147064

[20] Nikpay M Goel A Won HH Hall LM Willenborg C Kanoni S. A comprehensive 1,000 Genomes-based genome-wide association meta-analysis of coronary artery disease. Nat Genet. (2015) 47:1121–30. 10.1038/ng.3396 26343387 PMC4589895

[21] Nielsen JB Thorolfsdottir RB Fritsche LG Zhou W Skov MW Graham SE. Biobank-driven genomic discovery yields new insight into atrial fibrillation biology. Nat Genet. (2018) 50:1234–9. 10.1038/s41588-018-0171-3 30061737 PMC6530775

[22] Shah S Henry A Roselli C Lin H Sveinbjörnsson G Fatemifar G. Genome-wide association and Mendelian randomisation analysis provide insights into the pathogenesis of heart failure. Nat Commun. (2020) 11:163. 10.1038/s41467-019-13690-5 31919418 PMC6952380

[23] Auton A Brooks LD Durbin RM Garrison EP Kang HM Korbel JO. A global reference for human genetic variation. Nature. (2015) 526:68–74. 10.1038/nature15393 26432245 PMC4750478

[24] Bowden J Davey Smith G Haycock PC Burgess S. Consistent estimation in Mendelian randomization with some invalid instruments using a weighted median estimator. Genet Epidemiol. (2016) 40:304–14. 10.1002/gepi.21965 27061298 PMC4849733

[25] Zhao Q Wang J Hemani G Bowden J Small DS. Statistical inference in two-sample summary-data Mendelian randomization using robust adjusted profile score. Annals Statistics. (2018) 48:1742–69. 10.1214/19-AOS1866

[26] Verbanck M Chen CY Neale B Do R. Detection of widespread horizontal pleiotropy in causal relationships inferred from Mendelian randomization between complex traits and diseases. Nat Genet. (2018) 50:693–8. 10.1038/s41588-018-0099-7 29686387 PMC6083837

[27] Bowden J Del Greco MF Minelli C Davey Smith G Sheehan N Thompson J. A framework for the investigation of pleiotropy in two-sample summary data Mendelian randomization. Stat Med. (2017) 36:1783–802. 10.1002/sim.7221 28114746 PMC5434863

[28] Bowden J Davey Smith G Burgess S. Mendelian randomization with invalid instruments: effect estimation and bias detection through Egger regression. Int J Epidemiol. (2015) 44:512–25. 10.1093/ije/dyv080 26050253 PMC4469799

[29] Burgess S Thompson SG. Multivariable Mendelian randomization: the use of pleiotropic genetic variants to estimate causal effects. Am J Epidemiol. (2015) 181:251–60. 10.1093/aje/kwu283 25632051 PMC4325677

[30] Hoffmann TJ Choquet H Yin J Banda Y Kvale MN Glymour M. A large multiethnic genome-wide association study of adult body mass index identifies novel loci. Genetics. (2018) 210:499–515. 10.1534/genetics.118.301479 30108127 PMC6216593

[31] Yavorska OO Burgess S. MendelianRandomization: an R package for performing Mendelian randomization analyses using summarized data. Int J Epidemiol. (2017) 46:1734–9. 10.1093/ije/dyx034 28398548 PMC5510723

[32] Mouridsen SE Rich B Isager T. Diseases of the circulatory system among adult people diagnosed with infantile autism as children: a longitudinal case control study. Res Dev Disabil. (2016) 57:193–200. 10.1016/j.ridd.2016.07.002 27448332

[33] Tyler CV Schramm SC Karafa M Tang AS Jain AK. Chronic disease risks in young adults with autism spectrum disorder: forewarned is forearmed. Am J Intellect Dev Disabil. (2011) 116:371–80. 10.1352/1944-7558-116.5.371 21905805

[34] Dhaliwal KK Orsso CE Richard C Haqq AM Zwaigenbaum L. Risk Factors for unhealthy weight gain and obesity among children with autism spectrum disorder. Int J Mol Sci. (2019) 20:3285. 10.3390/ijms20133285 31277383 PMC6650879

[35] Stanish HI Curtin C Must A Phillips S Maslin M Bandini LG. Physical activity levels, frequency, and type among adolescents with and without autism spectrum disorder. J Autism Dev Disord. (2017) 47:785–94. 10.1007/s10803-016-3001-4 28066867 PMC5437850

[36] Egan AM Dreyer ML Odar CC Beckwith M Garrison CB. Obesity in young children with autism spectrum disorders: prevalence and associated factors. Child Obes. (2013) 9:125–31. 10.1089/chi.2012.0028 23485020

[37] Hagger-Johnson G Roberts B Boniface D Sabia S Batty GD Elbaz A. Neuroticism and cardiovascular disease mortality: socioeconomic status modifies the risk in women (UK Health and Lifestyle Survey). Psychosom Med. (2012) 74:596–603. 10.1097/PSY.0b013e31825c85ca 22753630

[38] Kranert M Benz ABE Shchetynska-Marinova T Hetjens S Liebe V Rosenkaimer S. Perception of atrial fibrillation in dependence of neuroticism. J Psychosom Res. (2020) 138:110225. 10.1016/j.jpsychores.2020.110225 32877820

[39] Riese H Rosmalen JG Ormel J Van Roon AM Oldehinkel AJ Rijsdijk FV. The genetic relationship between neuroticism and autonomic function in female twins. Psychol Med. (2007) 37:257–67. 10.1017/S0033291706009160 17094814

[40] Cukić I Bates TC. The association between neuroticism and heart rate variability is not fully explained by cardiovascular disease and depression. PLoS ONE. (2015) 10:e0125882. 10.1371/journal.pone.0125882 25951236 PMC4423941

[41] Tessier P Lelorain S Bonnaud-Antignac A. A comparison of the clinical determinants of health-related quality of life and subjective well-being in long-term breast cancer survivors. Eur J Cancer Care. (2012) 21:692–700. 10.1111/j.1365-2354.2012.01344.x 22471301

[42] Jebb AT Morrison M Tay L Diener E. Subjective well-being around the world: trends and predictors across the life span. Psychol Sci. (2020) 31:293–305. 10.1177/0956797619898826 32045327

[43] Shirai K Iso H Ohira T Ikeda A Noda H Honjo K. Perceived level of life enjoyment and risks of cardiovascular disease incidence and mortality: the Japan public health center-based study. Circulation. (2009) 120:956–63. 10.1161/CIRCULATIONAHA.108.834176 19720937

[44] Boehm JK Peterson C Kivimaki M Kubzansky LD. Heart health when life is satisfying: evidence from the Whitehall II cohort study. Eur Heart J. (2011) 32:2672–7. 10.1093/eurheartj/ehr203 21727096 PMC3205478

[45] Boehm JK Peterson C Kivimaki M Kubzansky L. A prospective study of positive psychological well-being and coronary heart disease. Health Psychol. (2011) 30:259–67. 10.1037/a0023124 21553969 PMC3165195

[46] Boehm JK Kubzansky LD. The heart's content: the association between positive psychological well-being and cardiovascular health. Psychol Bull. (2012) 138:655–91. 10.1037/a0027448 22506752

[47] Diener E Oishi S Tay L. Advances in subjective well-being research. Nat Hum Behav. (2018) 2:253–60. 10.1038/s41562-018-0307-6 30936533

[48] Wang K Ding L Yang C Hao X Wang C. Exploring the relationship between psychiatric traits and the risk of mouth ulcers using bi-directional mendelian randomization. Front Genet. (2020) 11:608630. 10.3389/fgene.2020.608630 33424931 PMC7793678

