# Supplementary figures and images for: Association of Autism Spectrum Disorder, Neuroticism, and Subjective Well-Being With Cardiovascular Diseases: A Two-Sample Mendelian Randomization Study
Source: Front Cardiovasc Med. 2021 Jun 11;8:676030. doi: 10.3389/fcvm.2021.676030 (PMC8225943; doi:10.3389/fcvm.2021.676030)

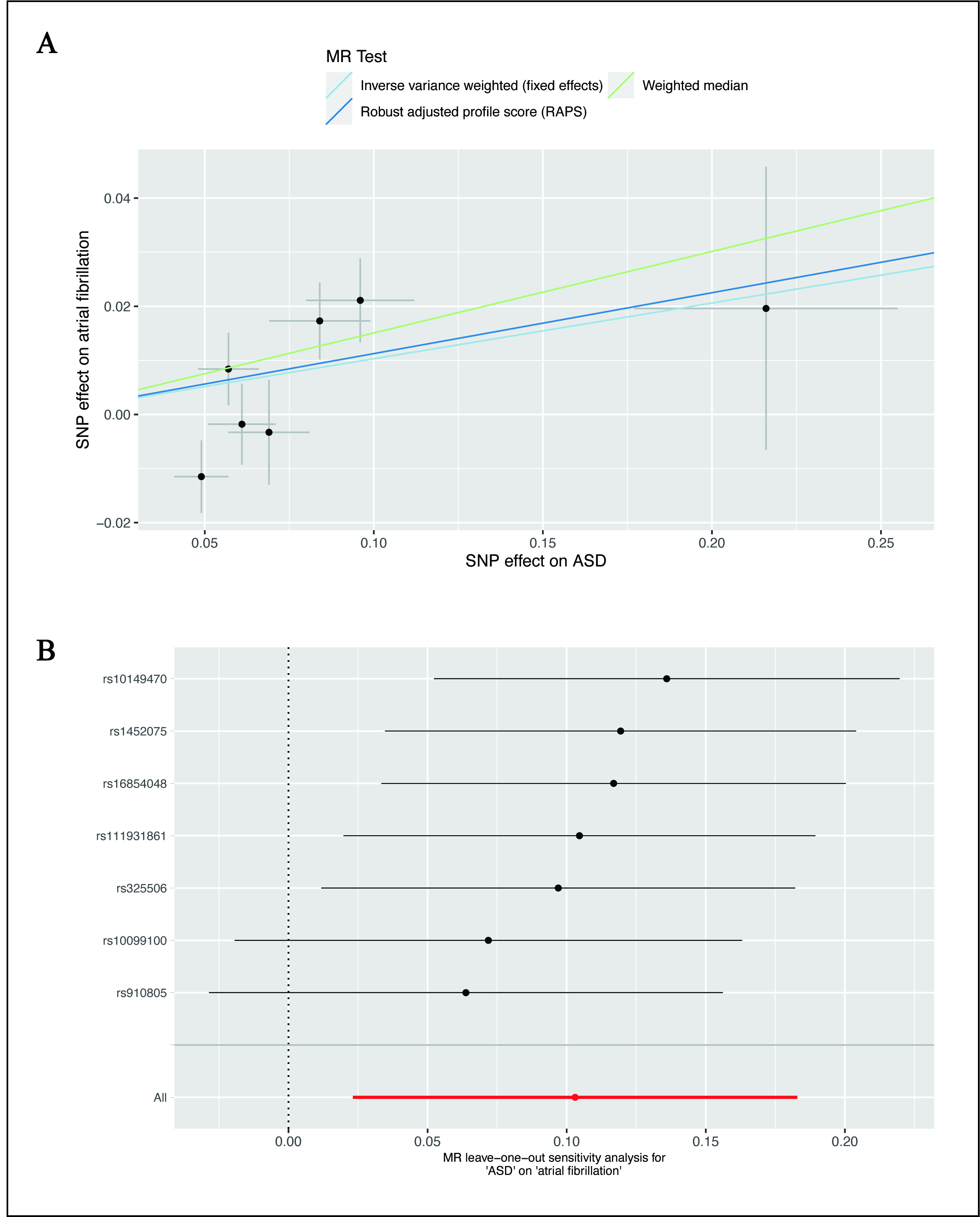

Supplement: Supplementary file 1 [file Presentation_1.zip › Supplementary Figure 1.tif]

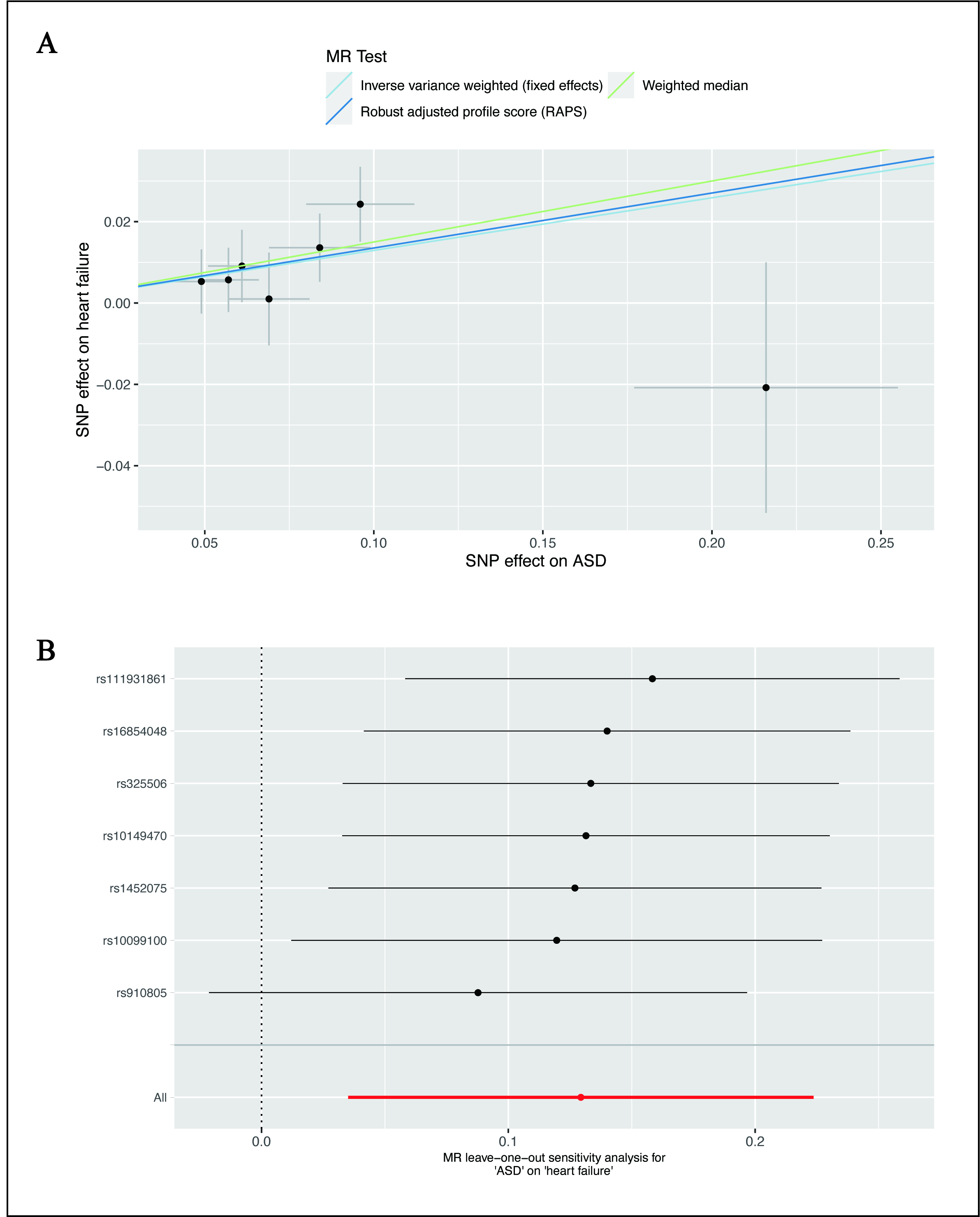

Supplement: Supplementary file 1 [file Presentation_1.zip › Supplementary Figure 2.tif]

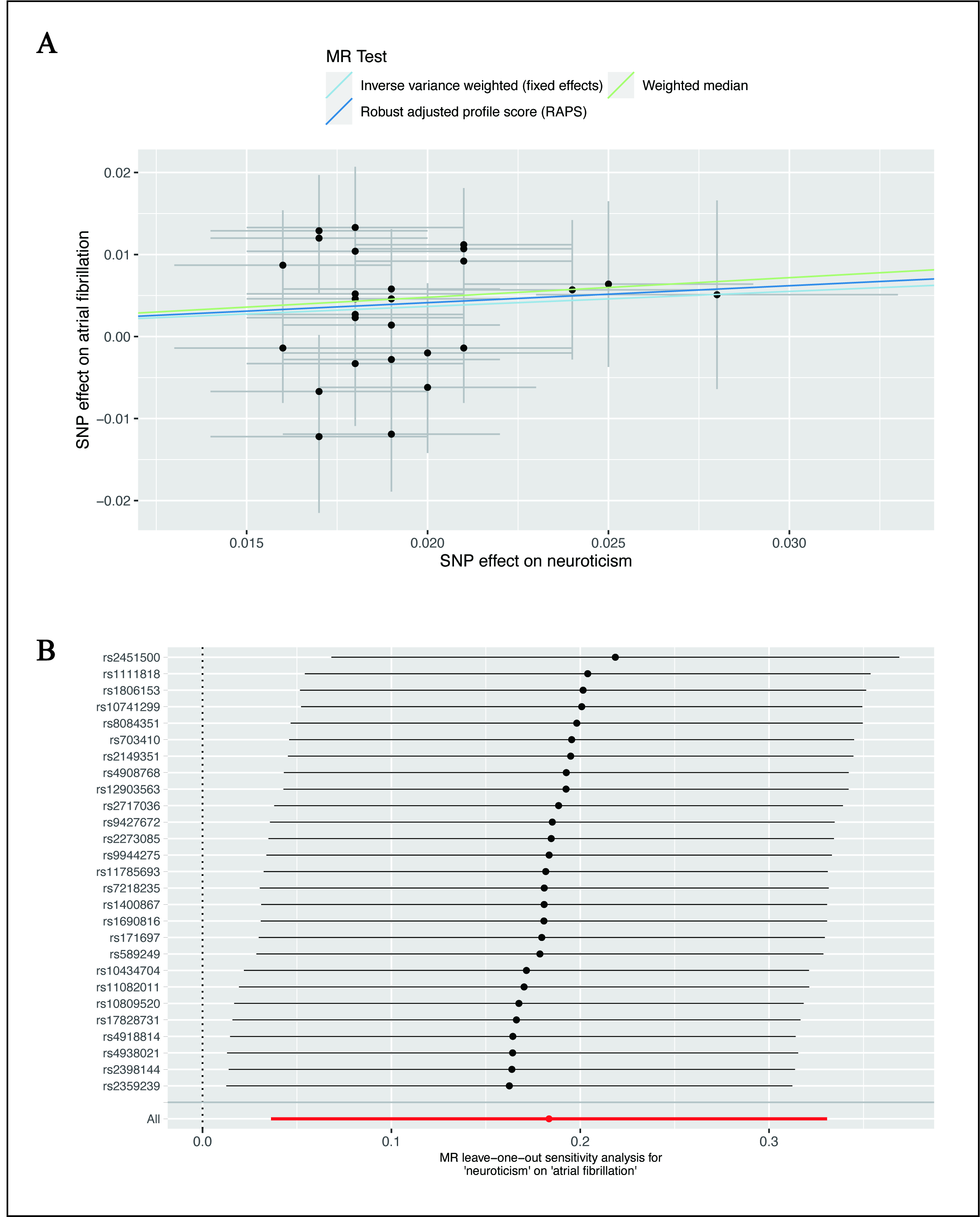

Supplement: Supplementary file 1 [file Presentation_1.zip › Supplementary Figure 3.tif]

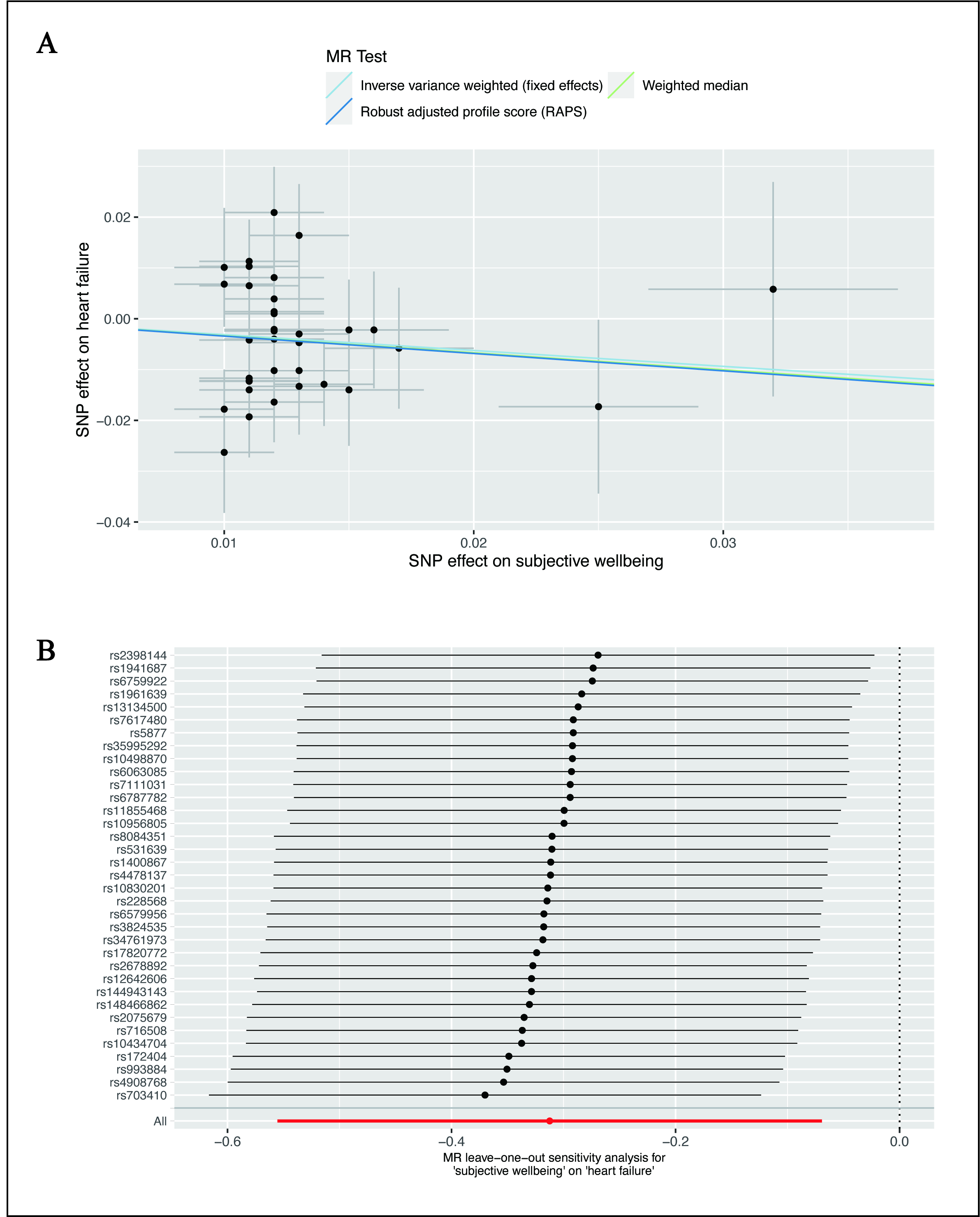

Supplement: Supplementary file 1 [file Presentation_1.zip › Supplementary Figure 4.tif]
