# Supplementary material for: Association of Autism Spectrum Disorder, Neuroticism, and Subjective Well-Being With Cardiovascular Diseases: A Two-Sample Mendelian Randomization Study
Source: Front Cardiovasc Med. 2021 Jun 11;8:676030. doi: 10.3389/fcvm.2021.676030 (PMC8225943; doi:10.3389/fcvm.2021.676030)
Supplement: Supplementary file 2 [file Table_1.DOCX]

Supplementary Material

# Supplementary Figures and Tables

## Supplementary Figures

**Supplementary Figure 1.** A) Scatter plot and B) leave-one-out analysis of autism spectrum disorder on atrial fibrillation

Abbreviations: MR, Mendelian randomization; SNP, single-nucleotide polymorphism; ASD, autism spectrum disorder.

**Supplementary Figure 2.** A) Scatter plot and B) leave-one-out analysis of autism spectrum disorder on heart failure

Abbreviations: MR, Mendelian randomization; SNP, single-nucleotide polymorphism; ASD, autism spectrum disorder.

**Supplementary Figure 3.** A) Scatter plot and B) leave-one-out analysis of neuroticism on atrial fibrillation

Abbreviations: MR, Mendelian randomization; SNP, single-nucleotide polymorphism

**Supplementary Figure 4.** A) Scatter plot and B) leave-one-out analysis of subjective wellbeing on heart failure

Abbreviations: MR, Mendelian randomization; SNP, single-nucleotide polymorphism.

## Supplementary Tables

**Supplementary Table 1:** Association of potential pleiotropic SNPs with cardiovascular risk factors searched in the Phenoscanner database

| **Exposure** | **SNP** | **Effect allele** | **Non-effect allele** | **Trait** | **Beta** | **P-value** |
| --- | --- | --- | --- | --- | --- | --- |
| ASD | rs2388334 | A | G | Body mass index | 0.015 | 2.97E-10 |
|  | rs11787216 | C | T | Weight | -0.013 | 1.72E-08 |
|  | rs1620977 | A | G | Body mass index | 0.024 | 1.07E-12 |
| Neuroticism | rs35991856 | A | C | Body mass index | 0.033 | 1.48E-20 |
|  |  |  |  | Diastolic blood pressure | 0.021 | 1.70E-08 |
|  | rs149866169 | A | T | Diastolic blood pressure | -0.021 | 1.76E-08 |
|  | rs10244364 | C | T | Smoking | 0.009 | 4.42E-12 |
|  | rs7107356 | A | G | Body mass index | 0.018 | 1.44E-13 |
|  |  |  |  | Systolic blood pressure | -0.017 | 1.62E-12 |
|  |  |  |  | Diastolic blood pressure | -0.014 | 1.03E-08 |
| Subjective wellbeing | rs12187898 | C | T | Body mass index | 0.014 | 1.96E-08 |
|  | rs10920678 | A | G | Body mass index | 0.018 | 8.03E-14 |
|  | rs10514301 | C | T | Body mass index | -0.033 | 1.10E-18 |
|  | rs2243616 | T | G | High cholesterol | -0.006 | 3.20E-13 |
|  | rs10953620 | A | C | Body mass index | -0.014 | 1.87E-08 |

The reported beta and P-values were obtained from PhenoScanner v2 on February 18, 2021. Abbreviations: SNP, single-nucleotide polymorphism; ASD, autism spectrum disorder.

**Supplementary Table 4.** Power calculation for two-sample Mendelian randomization analyses of three psychiatric traits on four cardiovascular diseases.

| **Exposure** | **Outcome** | **Variance Explained by the Instruments (*R*^2^)** | ***Required ORs* (power = 80%)** | ***Estimated ORs*** |
| --- | --- | --- | --- | --- |
| ASD | CAD | 1.50E-02 | 1.117 | 0.997 |
|  | MI |  | 1.130 | 0.993 |
|  | AF |  | 1.096 | 1.109 |
|  | HF |  | 1.108 | 1.138 |
| Neuroticism | CAD | 3.98E-03 | 1.235 | 1.111 |
|  | MI |  | 1.260 | 1.179 |
|  | AF |  | 1.187 | 1.201 |
|  | HF |  | 1.210 | 1.029 |
| Subjective wellbeing | CAD | 2.18E-03 | 0.733 | 0.886 |
|  | MI |  | 0.698 | 0.823 |
|  | AF |  | 0.754 | 0.898 |
|  | HF |  | 0.723 | 0.732 |

Abbreviations: ASD, autism spectrum disorder; CAD, coronary artery disease; MI, myocardial infarction; AF, atrial fibrillation; HF, heart failure; OR, odds ratio.

**Supplementary Table 5:** Results of the Mendelian randomization analyses of autism spectrum disorder, neuroticism, and subjective wellbeing on atrial fibrillation and heart failure by adopting genetic variants at a threshold of P < 1×10^-6^

| **Exposure** | **Outcome** | **Methods** | **SNPs** | **OR (95% CI)** | **P-value** |
| --- | --- | --- | --- | --- | --- |
| ASD | AF | IVW (fixed effects) | 16 | 1.055 (1.005-1.108) | 0.028 |
|  |  | Weighted median | 16 | 1.021 (0.951-1.096) | 0.589 |
|  |  | MR-RAPS | 16 | 1.047 (0.98-1.118) | 0.192 |
|  | HF | IVW (fixed effects) | 17 | 1.123 (1.058-1.191) | 2.43E-04 |
|  |  | Weighted median | 17 | 1.109 (1.023-1.203) | 0.021 |
|  |  | MR-RAPS | 17 | 1.126 (1.056-1.201) | 5.03E-04 |
| Neuroticism | AF | IVW (fixed effects) | 78 | 1.058 (0.959-1.166) | 0.260 |
|  |  | Weighted median | 78 | 1.127 (0.980-1.295) | 0.094 |
|  |  | MR-RAPS | 78 | 1.077 (0.971-1.194) | 0.162 |
| Subjective wellbeing | HF | IVW (fixed effects) | 73 | 0.777 (0.648-0.932) | 0.007 |
|  |  | IVW (random effects) | 73 | 0.777 (0.620-0.974) | 0.029 |
|  |  | Weighted median | 73 | 0.711 (0.536-0.945) | 0.019 |
|  |  | MR-RAPS | 73 | 0.759 (0.593-0.972) | 0.029 |

Abbreviations: SNPs, single-nucleotide polymorphisms; OR, odds ratio; CI, confidence interval; ASD, autism spectrum disorder; AF, atrial fibrillation; HF, heart failure; IVW, inverse-variance weighted; MR-RAPS, Mendelian randomization-robust adjusted profile score.

**Supplementary Table 6:** Results of potential pleiotropy evaluation by adopting genetic variants at a threshold of P < 1×10^-6^

| **Exposure** | **Outcome** | **SNPs** | **Cochran's Q statistic** | **Cochran's Q** | **MR-Egger intercept** | **MR-PRESSO global test** | **MR-PRESSO global test** |
| --- | --- | --- | --- | --- | --- | --- | --- |
|  |  |  |  | **P** | **P** |  | **P** |
| ASD | AF | 18 | 22.684 | 0.160 | 0.936 | 25.880 | 0.163 |
|  | HF | 17 | 11.177 | 0.798 | 0.743 | 12.773 | 0.790 |
| Neuroticism | AF | 78 | 75.033 | 0.542 | 0.073 | 76.941 | 0.535 |
| Subjective wellbeing | HF | 73 | 111.274 | 0.002 | 0.534 | 114.315 | 0.002 |

Abbreviations: SNP, single-nucleotide polymorphism; MR-Egger, Mendelian randomization-Egger; MR-PRESSO, Mendelian randomization pleiotropy residual sum and outlier; ASD, autism spectrum disorder; AF, atrial fibrillation; HF, heart failure.

**Supplementary Table 2:** Genetic association estimates for the association between three psychiatric traits and cardiovascular diseases, and multivariable Mendelian randomization analyses adjusted for body mass index and blood pressure measurements

Abbreviations: SNP, single-nucleotide polymorphism; EA, effect allele; NEA, non-effect allele; EAF, effect allele frequency; R^2^, percentage of the variation explained by the SNPs, calculated as R^2^ = 2 × minor allele frequency × (1 − minor allele frequency) × (β / SD) ^2^; F, F statistic; SE, standard error of Beta; ASD, autism spectrum disorder; CAD, coronary artery disease; MI, myocardial infarction; AF, atrial fibrillation; HF, heart failure; BMI, body mass index; SBP, systolic blood pressure; DBP, diastolic blood pressure.

**Supplementary Table 3:** Genetic association estimates for the association between three psychiatric traits and cardiovascular diseases with SNPs adopted at a threshold of P value < 1×10^-6^ Abbreviations: SNP, single-nucleotide polymorphism; EA, effect allele; NEA, non-effect allele; EAF, effect allele frequency; SE, standard error of Beta; ASD, autism spectrum disorder; AF, atrial fibrillation; HF, heart failure; BMI, body mass index. The SNPs associated with ASD at the level of genome-wide significance arose from the main ASD scan, the combined analysis with the follow-up sample, and three MTAG analyses. Only the full summary statistics for the main ASD scan were available, thus, the SNPs at 5×10^-8^ ≤ P < 1×10^-6^ were selected from the summary-level data for the main ASD scan. *: The SNPs not available in the outcome datasets were replaced by proxy SNPs (in brackets, r^2^ > 0.8).
